# Exploring the anti‐inflammatory ingredients and potential of golden buckwheat (*Fagopyrum dibotrys*) on the TLR4/NLRP3 pathway in acute lung injury

**DOI:** 10.1002/fsn3.4193

**Published:** 2024-04-26

**Authors:** Yingfan Hu, Xiaomin Liu, Yu Song, Yan Zhang, Wei Li, Lele Zhang, Anqi Wang, Qian Su, Zhiyong Yang, Liang Zou

**Affiliations:** ^1^ School of Preclinical Medicine Chengdu University Chengdu Sichuan China; ^2^ State Key Laboratory of Quality Research in Chinese Medicine University of Macau Macao China; ^3^ Key Laboratory of Coarse Cereal Processing of Ministry of Agriculture and Rural Affairs Chengdu University Chengdu Sichuan China; ^4^ Clinical Medical College and Affiliated Hospital of Chengdu University Chengdu Sichuan China

**Keywords:** acute lung injury, anti‐inflammatory, *Fagopyrum dibotrys*, golden buckwheat, TLR4/NLRP3

## Abstract

Golden buckwheat, also called *Fagopyrum dibotrys* (D. Don) H. Hara, is a plant of the genus Buckwheat in the buckwheat family. The aim of this study was to screen the bioactive ingredients of golden buckwheat extract and investigate the protective effect on acute lung injury (ALI). The ethyl acetate extract (EAE) was identified as the active fraction in LPS‐induced RAW264.7 cells, with gallic acid, proanthocyanidin B2, and epicatechin at 0.0563%, 0.3707%, and 0.3868%, respectively. At the same time, 20 compounds (mainly flavonoids and organic acids) were identified by UPLC‐Q‐Exactive Orbitrap‐HRMS in EAE. Furthermore, the EAE reduced lung histopathology scores in mice with ALI, decreased the dry‐to‐wet weight ratio of lung tissue, and significantly inhibited the concentrations of IL‐1β, TNFα, and IL‐6 in bronchoalveolar lavage fluid (BALF). It also reduced the number of leukocytes, decreased the activity of MPO in lung tissue, and inhibited the levels of TLR4/NLRP3 pathway mRNA and protein in lung tissue. Our study indicated that golden buckwheat as a source of functional food prevents or treats associated lung diseases by modulating the activation of the TLR4/NLRP3 signaling pathway.

## Graphical abstract

In this work, we screened and characterized the anti‐inflammatory active components of *Fagopyrum dibotrys* (D. Don) H. Hara and investigated their protective mechanism of action against lipopolysaccharide (LPS)‐induced acute lung injury (ALI) in mice based on the TLR4/NLRP3 signaling pathway.

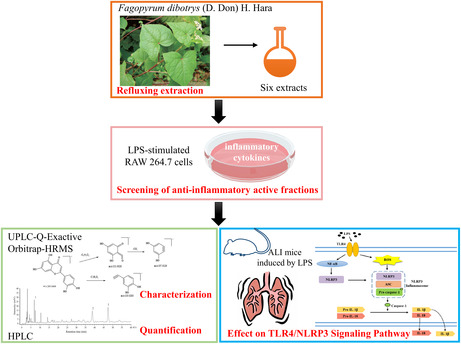

## 1 INTRODUCTION

Acute lung injury (ALI) is a severe respiratory disorder recognized as causing inflammation and damage to the alveoli or small air sacs, ultimately resulting in diffuse damage to the entire lungs (Long et al., 2022). The causes of ALI may include infection, trauma, sepsis, aspiration of gastric contents, and other types of injury to the lungs. It can lead to respiratory failure and other complications, and is often a precursor to the grosser condition of acute respiratory distress syndrome (ARDS), which can lead to fulminant respiratory insufficiency and even death (Meyer et al., 2021). Studies have also shown that a significant minority of inpatient with coronavirus disease 2019 (COVID‐19) developed complications of ARDS (Swenson & Swenson, 2021). Given the high mortality rate of ARDS, early prevention and treatment management are crucial for improving disease prognosis and increasing recovery opportunities.

The pathogenesis of ALI involves a number of factors, including infection, inflammation, and oxidative stress. Lipopolysaccharide (LPS), as an important component that causes inflammation, is a molecule found in the membrane of Gram‐negative bacteria. LPS can cause various infections, involving life‐threatening pneumonia and sepsis, and has become a common model inducer for preparing ALI (Long et al., 2022). When LPS is released into the lungs in response to infection or injury, Toll‐like receptor 4 (TLR4) specifically distinguishes LPS, bringing about the formation of a signaling complex that activates a cascade of downstream signals. Recent studies suggested that the NLRP3 inflammasome, a multiprotein polymer that plays an important role in innate immune response, might also be excited by TLR4 in the presence of LPS (Sun & Li, 2022). Once activated, NLRP3 inflammatory leads to the excitation of caspase‐1, which in turn stimulates the NLRP3 inflammatory receptor and caspase‐1 precursors. The caspase‐1 splits pro‐interleukin (IL)‐1β and pro‐IL‐18. Activated IL‐1β and IL‐18 are liberated and further aggravate the inflammatory response and contribute to ALI. The stimulation of TLR4 and NLRP3 inflammasome by LPS can also disrupt the balance of oxidative stress by producing reactive oxygen species (ROS), which can engender direct tissue injury and promote further inflammation (Wang et al., 2020). Overall, the participation of TLR4/NLRP3 in the pathogenesis of ALI highlights the importance of innate immunity and inflammation in the development of this condition. Targeting these pathways may provide therapeutic possibilities for the therapy of ALI caused by LPS and other inflammatory mediators.

*Fagopyrum dibotrys* (D. Don) H. Hara (also called golden buckwheat) is a perennial herb of the genus buckwheat in the family Polygonaceae (Zhang, He, et al., 2021). As a medicinal food homology plant, it has been commonly consumed in China, India, Japan, Canada, Italy, and other countries, and has been authenticated as a functional food by the National Health Commission of the People's Republic of China. Golden buckwheat has high nutritional value and is rich in active ingredients such as polyphenols, flavonoids, and amino acids. As a nutrient‐rich food source, it can be used in various recipes, including as a substitute for rice or as an ingredient for pancakes and bread. Otherwise, due to the ease of cultivation features, golden buckwheat has broad application prospects in green animal husbandry (Xiong et al., 2023). In addition, golden buckwheat is also an important herbal medicine, which has a vast history of use in traditional medicine, particularly in China. It is considered to have a series of health advantages, including alleviating inflammation, alleviating cholesterol levels, and improving digestive function. Pharmacological studies have shown that golden buckwheat had the inhibition of lung infection and inflammatory response, improving lung fibrosis, and repairing lung tissue (Zhang, He, et al., 2021). As a common Chinese medicine used for the clinical treatment of respiratory diseases, golden buckwheat tablets (capsules) were selected as one of the recommended drugs for the prevention and treatment of influenza, new coronavirus infection, hand, foot and mouth disease (Cheng et al., 2021). However, the pharmacodynamic basis of golden buckwheat in the treatment of ALI and its effect on TLLR4/NLRP3 is unclear.

Therefore, this time, the anti‐inflammatory activities of total ethanol extract and different polar fractions of golden buckwheat were investigated based on LPS‐induced RAW 264.7 cells, and the components and pharmacological active substance contents of the selected optimal anti‐inflammatory active parts were analyzed using UPLC‐Q‐Exactive Orbitrap‐HRMS. Furthermore, based on the TLR4/NLRP3 signaling pathway, the active mechanism of golden buckwheat in the combating ALI was discussed.

## 2 MATERIALS AND METHODS

### 2.1 Chemicals and reagents

Golden buckwheat pieces were obtained from Anhui Daoyuantang Chinese Herbal Pieces Co., Ltd. (Bozhou, China). LPS was acquired from Sigma‐Aldrich Co. LLC (St. Louis, USA). Dexamethasone acetate tablets were supplied by Chengdu First Pharmaceutical Co. Ltd. (Chengdu, China). The supplier of epicatechins (≥98%), proanthocyanidin B2 (≥98%), gallic acid (≥98%), and berberine (≥98%) was Chengdu MUST Bio‐Technology Co., Ltd. (Chengdu, China). Quercetin (≥98%) and rutin (≥98%) were provided by Chengdu Herbpurify Co., Ltd. (Chengdu, China). Hypericin (94.9%) and protocatechuic acid (97.7%) were acquired from the National Institute for Food and Drug Control (Beijing, China). Luteolin (≥98%) was from Beijing Solarbio Science & Technology Co., Ltd. (Beijing, China). Cell counting kit‐8 (CCK‐8) was provided by Boster Biological Technology Co., Ltd. (Wuhan, China). Myeloperoxidase (MPO) kit was purchased from Nanjing Jiancheng Bioengineering Institute (Nanjing, China). The ELISA kits of inflammatory cytokines were purchased from MultiSciences Biotech Co., Ltd. (Hangzhou, China) and ExCell Biotechnology Co., Ltd. (Shanghai, China). The quantitative Real‐time PCR‐related reagents were supplied by Wuhan Servicebio Technology Co., Ltd. (Wuhan, China). NLRP3 and ASC antibodies were purchased from Abcam (Cambridge, USA) and Santa Cruz Biotechnology (Santa Cruz, USA), respectively. The antibodies (caspase‐1 and IL‐1β) used were provided by Cell Signaling Technology, Inc. (Danvers, USA). GAPDH and secondary antibodies were presented by Zen BioScience (Chengdu, China).

### 2.2 Preparation of golden buckwheat extracts

Golden buckwheat pieces were crushed and then extracted three times with an 8‐fold amount of 95% ethanol reflux for 2 h each. The filtrate was combined and evaporated with a rotary evaporator to obtain the extractum (paste). The extractum was dissolved with water and repeatedly extracted with petroleum ether, chloroform, ethyl acetate, and n‐butanol until colorless. We collected the extracted solution separately and concentrated the recovered solvent under reduced pressure. According to this method, the total extract (TE), petroleum ether extract (PEE), chloroform extract (CE), ethyl acetate extract (EAE), n‐butanol extract (BE), and aqueous extract (AE) were attained.

### 2.3 Cell culture and CCK‐8 assay

The RAW 264.7 mouse macrophage cell line was supplied by Cell Bank/Stem Cell Bank (Shanghai, China). Cells were grown in DMEM containing penicillin streptomycin solution and fetal bovine serum, and housed at the cell culture incubator (37°C, 5% CO_2_, and 95% humidity). Cells in the log phase were plated in 96‐well plates. After incubation for 24 h, the cells were treated with different concentrations of each extract for another 24 h. Subsequently, after 40 min of incubation with a 10% CCK‐8 solution. At a wavelength of 450 nm, the optical density of the absorbance was determined.

### 2.4 Screening of anti‐inflammatory active fractions

Logarithmic macrophages were seeded in 24‐well plates for 24 h, and then incubated at each extractive fraction at the same concentration for 30 min except for the control and model group. All groups were co‐cultured with LPS (1 μg/mL) in the CO_2_ incubator for another 24 h with the exception of the control group. The cell supernatant was received and the secretion of NO, IL‐6, and IL‐1β in it was measured as described in the kit instructions (Taherzadeh et al., 2022).

### 2.5 Determinations of the active component

The instrument for content determination was the HPLC system (Shimadzu Industrial Systems Co., Ltd.). The column was INSTRU RXB‐C_18_ (4.6 mm × 250 mm, 5 μm). The mobile phase was acetonitrile (A): 0.1% formic acid (B) at 1 mL/min. The elution gradients were as below: 0–5 min, 5% (A); 5–10 min, 5%–7.5% (A); 10–70 min, 7.5%–10% (A); 70–80 min, 10%–5% (A); 80–85 min, 5% (A). The test volume was 10 μL at a column temperature of 35°C. The detection wavelength was 280 nm. Validation experiments were performed to authenticate the developed method. Specific validation parameters including specificity, linearity, precision, reproducibility, stability, and recovery were assessed.

### 2.6 Component identification by UPLC‐Q‐Exactive Orbitrap‐HRMS

The core instrument for component analysis was UPLC‐Q‐Exactive Orbitrap‐HRMS (Thermo Fisher Scientific, Inc., USA). The column was Thermo Scientific Accucore™ C_18_ (2.1 mm × 100 mm, 2.6 μm). The mobile phases used were the same as the part of 2.5. The elution gradients were carried out as listed below: 0–5 min, 5% (A); 5–15 min, 5%–20% (A); 15–25 min, 20%–40% (A); 25–40 min, 40%–80% (A); 40–50 min, 80%–95% (A); 50–55 min, 95%–5% (A). With the flow rate of 0.3 mL/min, the column temperature was 30°C and the injection volume was 3 μL.

The mass spectrometry (MS) analysis was performed using an electrospray ion source (ESI) with positive and negative ion switch scanning detection mode. The spray voltage was 3.5 kV. The temperature of the auxiliary gas heater was set to 350°C, and the temperature of the ion transport tube was set to 320°C. The gas flow rate of the sheath and auxiliary were 35 Arb and 10 Arb, individually. The scanning mode is primary MS full scan combined with automatic trigger secondary MS scan mode (full MS/dd‐MS^2^) with parameters 70,000 (full MS)/17,500 (dd‐MS^2^) scanning scope of *m/z* 100–1000. Collision energies were 20, 40, and 60 eV.

According to the accurate relative molecular mass of the primary MS, the peak extraction of the obtained total ion flow map (TIC) was carried out by Xcalibur™ software and matched with zCloud, Mass Bank, and other databases to preliminarily predict the molecular information. The chemical composition was further analyzed and identified according to the secondary fragment ion information provided by the reference substance, relevant references, and database.

### 2.7 Establishment of ALI model and experimental design

SPF male Kunming mice (weight: 30–35 g, age: 3–8 weeks) were acquired from Chengdu Dossy Experimental Animals Co., Ltd. (approval NO. SCXK [Chuan] 2020‐030). All mice were allowed to drink and eat, settled in a 12‐h light/dark cycle, and acclimatized to sensible feeding for 7 days prior to the study. They were divided into six treatment groups: control, LPS, dexamethasone (DXMS 2 mg/kg), and three doses of ethyl acetate extract (EAE 500, 250, and 125 mg/kg). Mice in DXMS and EAE groups were given intragastric administration with the respective drug, while the mice in control and LPS groups were treated with identical amounts of 0.5% CMC‐Na, once a diurnal for 1 week. After the final treatment 1 h, the mice were anesthetized in all groups except the control group. LPS solution (2.5 mg/kg) was dropped into the nose and mice were kept in an upright position and allowed to breathe freely into the trachea into the lungs. After 6 h of LPS treatment, the mice were sacrificed, and the bronchoalveolar lavage fluid (BALF) and lung tissue were sampled at once.

### 2.8 Histopathological examination of lung tissues

Fresh mouse lung tissue was fixed in a fixative for 24 h. After fixation, the tissue was dehydrated through a graded concentration of alcohol and inserted in paraffin. Specimens were cut into sections of approximately 3 μm sections, colored with hematoxylin–eosin (HE), sealed in clear plastic and photographed under a light microscope (200×).

### 2.9 Lung tissue wet/dry weight ratio (W/D)

After each mouse was sacrificed, the left lobe of it was excised. Lung samples were rinsed in saline solution, then the surface water was blotted off with filter paper and weighed (W). Lung tissue was then oven‐dried at 80°C for 72 h and reweighed (D). The ratio was evaluated to quantify the extent of edema in the lung tissue.

### 2.10 Detection of inflammatory cytokines and WBC in BALF

The lung was ligated and the windpipe was exposed for lavage. The windpipe was intubated with an intravenous needle, and PBS solution (500 μL) was injected into the trachea and retained for 10 s, then withdrawn and repeated three times. The lavage fluid was combined and centrifuged (800*g*, 10 min, 4°C). The upper liquid was removed and assayed for TNF‐α, IL‐6, and IL‐1β in the alveolar lavage solution according to the kit instructions (Askari et al., 2018). The bottom precipitate was resuspended with 500 μL of PBS solution and counted on a fully automated hematology analyzer to measure the number of WBC.

### 2.11 Measurement of MPO activity in the lung

Lung tissue was homogenized and centrifuged at 4°C and prepared for the detection of MPO activity. The measurement was performed by MPO kit, following strict instructions.

### 2.12 Analysis of real‐time qPCR

The lung tissue was dissected into tiny sections and placed in a pre‐cooled homogenizer to which RNA extract was remixed to lyse the homogenate. The upper phase was collected by centrifuging at 12,000 *g* for 10 min to separate the tissue fragments. Subsequently, the total RNA is isolated and the cDNA template is synthesized as described in the kit. Then, follow the guide of the RT‐qPCR kit to detect the levels of the target genes TLR4, NLRP3, ASC, caspase‐1, IL‐1β, IL‐18, and GAPDH messenger RNA (mRNA). The quantitative primer sequence was TLR4: (F) 5′‐TGAGGACTGGGTGAGAAATGAGC‐3′, (R) 5′‐CTGCCATGTTTGAGCAATCTCAT‐3′; NLRP3: (F) 5′‐TAAGAACTGTCATAGGGTCAAAACG‐3′, (R) 5′‐GTCTGGAAGAACAGGCAACATG‐3′; ASC: (F) 5′‐CATCCTGGACGCTCTTGAAAAC‐3′, (R) 5′‐CCATAGCCTTCTCGCAGTTGC‐3′; caspase‐1: (F) 5′‐GGCTGACAAGATCCTGAGGG‐3′, (R) 5′‐TAGGTCCCGTGCCTTGTCC‐3′; IL‐1β: (F) 5′‐GCATCCAGCTTCAAATCTCGC‐3′, (R) 5′‐TGTTCATCTCGGAGCCTGTAGTG‐3′; IL‐18: (F) 5′‐TGAAGTAAGAGGACTGGCTGTGA‐3′, (R) 5′‐TTGGCAAGCAAGAAAGTGTCC‐3′; GAPDH: (F) 5′‐CCTCGTCCCGTAGACAAAATG‐3′, (R) 5′‐TGAGGTCAATGAAGGGGTCGT‐3′; GAPDH was used as the housekeeper and the target gene levels were detected according to the ^∆∆^CT method.

### 2.13 Western blot test

Lung tissue samples were collected and further processed by lysing them with RIPA buffer containing protease inhibitors. This process yielded a supernatant that contained the proteins of interest. After quantification of the protein concentration by BCA, equal volumes of the protein samples were separated by SDS–PAGE and transferred onto a PVDF membrane through electrotransfer. To prevent non‐specific conjugation, the PVDF membranes were obstructed using 5% skimmed milk powder for 2 h. Following the blocking step, the membranes were swayed with 10 min TBST three times. Subsequently, they were incubated overnight at 4°C with the corresponding primary antibody. The primary antibodies include NLRP3 (Cat. #: ab270449), ASC (Cat. #: sc‐514414), caspase‐1 (Cat. #: 3866), IL‐1β (Cat. #: 83186), and GAPDH (Cat. #: 380626). Following another round of washing with TBST, the membranes were exposed to secondary antibody (Cat. # 511103 and 511203) for 1 h. Finally, bands were detected using chemiluminescence and the measured with ImageJ.

### 2.14 Data analyses

The results of the experiment were presented as $\bar{x}\pm s$. Analysis of variance (ANOVA) was used for comparisons between multiple groups and *t*‐test was used to quantify the difference between the mean (average) of a variable from up to two samples (datasets). The data were classified as statistically significant when *p* < .05.

## 3 RESULTS

### 3.1 Effect of golden buckwheat extracts on cell viability and inflammatory factors production in RAW 264.7 cells

**FIGURE 1:**
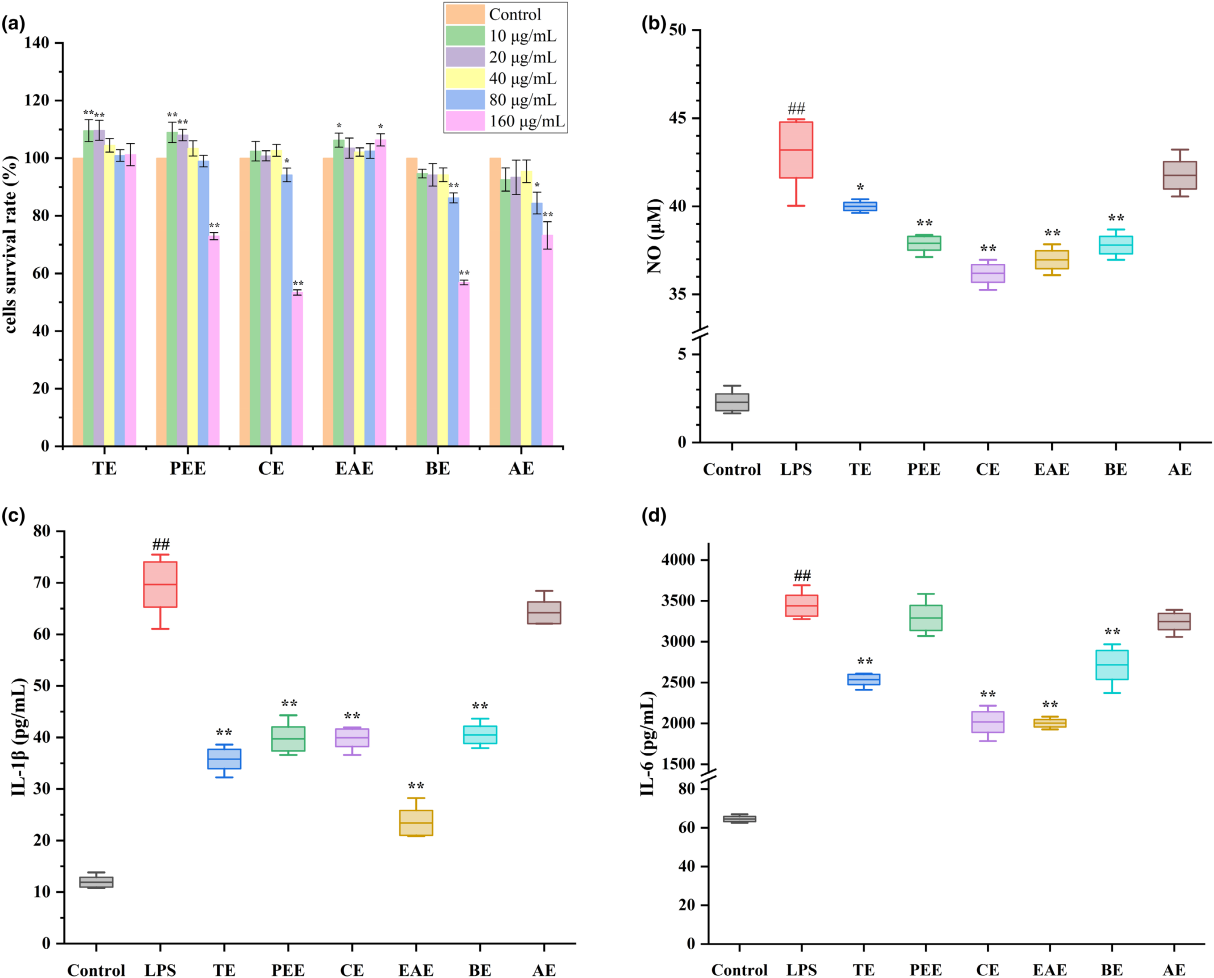
Effect of different concentrations of extracts on cells survival rate (a) and the same concentration (40 μg/mL) of extracts on the NO (b), IL‐1β (c), and IL‐6 (d) induction in LPS‐stimulated RAW 264.7 cells. AE, aqueous extract group; BE, n‐butanol extract group; CE, chloroform extract group; Control, control group; EAE, ethyl acetate extract group; LPS, LPS positive group; PEE, petroleum ether extract group; TE, total extract group. ^#^.01 ≤ *p* < .05 and ^##^ *p* < .01 versus control group; *.01 ≤ *p* < .05 and **p < .01 versus LPS group.

The impact of different concentrations of golden buckwheat extracts on the cell proliferation of RAW 264.7 cells is shown in Figure 1a. The results showed that when administered at concentrations below 40 μg/mL, the six extracts of golden buckwheat had no impact on the survival of RAW 264.7 cells. BE and AE exhibited a pronounced suppressive effect on the cell proliferation at a concentration of 80 μg/mL (*p* < .01). Similarly, PEE and CE showed a significant reduction in cell viability at a dose of 160 μg/mL (*p* < .01), while the TE and EAE still had no inhibitory effect on cell proliferation. The findings indicated that co‐incubation of the six golden buckwheat extracts with RAW 264.7 cells at a dose of 40 μg/mL for 24 h had no impact on cell viability. Therefore, to screen the anti‐inflammatory active fraction of golden buckwheat, a dose of 40 μg/mL was determined.

As depicted in Figure 1b–d, the levels of NO, IL‐1β, and IL‐6 in the cell supernatant exhibited a significant increase (*p* < .01) upon stimulation of RAW 264.7 cells with LPS. However, upon administration of 40 μg/mL of the extracts, TE, CE, EAE, and BE exhibited a noteworthy reduction in the secretion of NO, IL‐1β, and IL‐6. Additionally, PEE significantly lowered the concentration of NO and IL‐1β, while AE did not exhibit a significant effect. These findings indicated that all five extracts of golden buckwheat, with the exception of AE, possessed remarkable anti‐inflammatory activity. Compared to other extracts, both EAE and CE exhibited more potent inhibition of NO and IL‐6 secretion. Additionally, EAE demonstrated stronger inhibition of IL‐1β secretion compared to CE. Given its lower cytotoxicity and higher potency, EAE was identified as the active fraction and used in subsequent experiments.

### 3.2 Determinations of three active components of EAE based on HPLC

**FIGURE 2:**
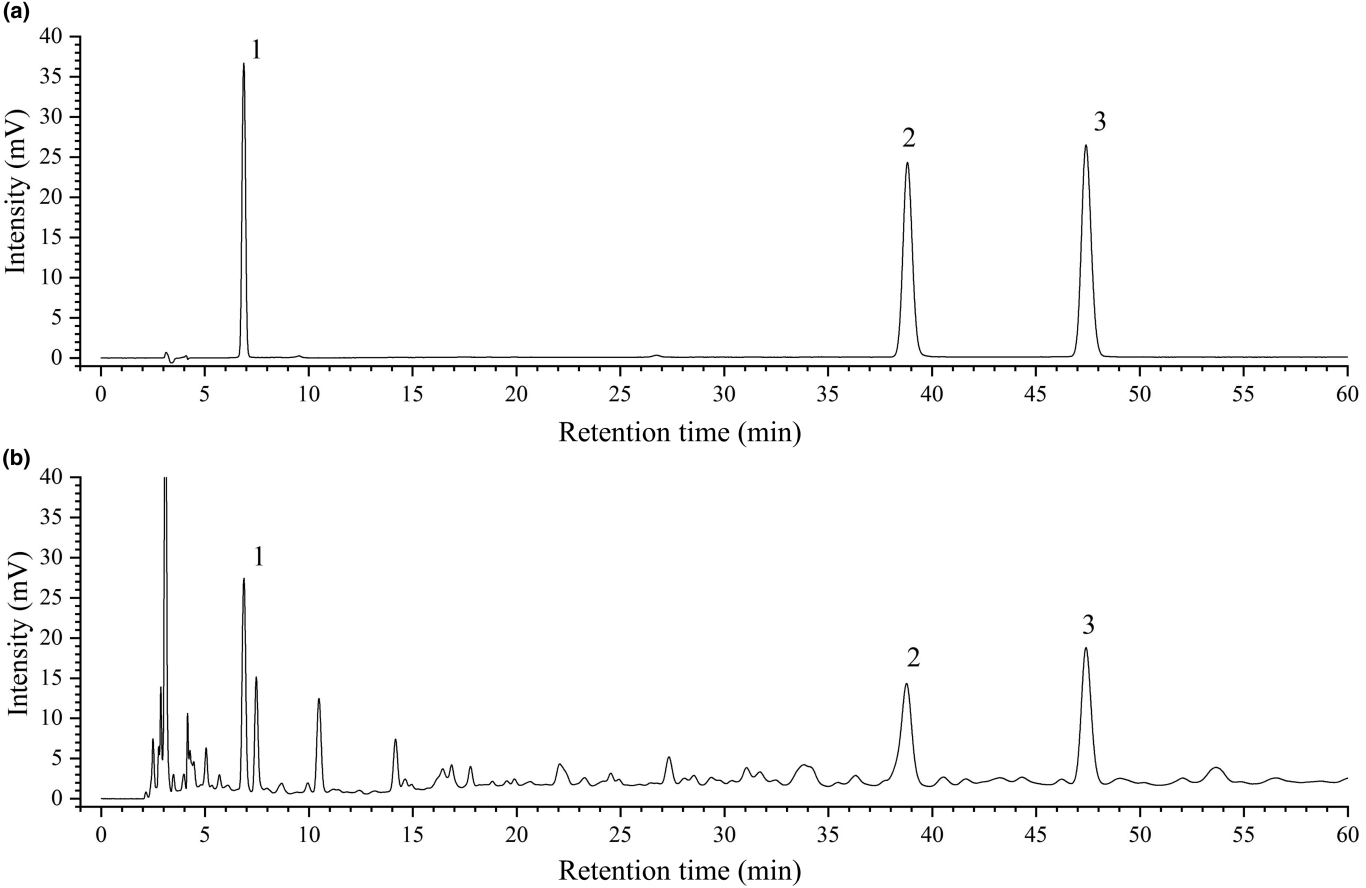
HPLC chromatogram of mixed reference solution (a) and sample solution (b). (1) gallic acid; (2) proanthocyanidin B2; and (3) epicatechin.

The EAE sample was pre‐treated and analyzed as described above for HPLC method validation, and specific validation parameters were determined. As shown in Figure 2, the method was able to separate and accurately measure the peaks of interest, indicating the specificity of the method. The chromatographic peaks of each component did not interfere with each other, nor did the methanol solution. The linear response was evaluated using standards at six concentrations. The regression correlation coefficients of gallic acid, proanthocyanidin B2, and epicatechin were 0.9996, 0.9994, and 0.9994, respectively, indicating that the linearity of the calibration curves was satisfactory within the range of concentrations considered to be appropriate for quantitative analysis. The precision of the chromatographic method was tested by checking the six replicate injections. The RSD values were <2%, demonstrating the good precision of the analytical method. The repeatability of the method was studied by preparing six samples in parallel, with RSD values ranging from 0.6% to 1.23%, which showed that the repeatability of tested samples was good. The stability of the gallic acid, proanthocyanidin B2, and epicatechin in the sample solution was tested. Samples were injected into the HPLC system at different times throughout the day. Upon inspection, the chromatograms of the stored samples exhibited no changes and did not reveal any additional peaks in comparison to the chromatograms of the freshly prepared samples. The sample solutions remained stable for a duration of 24 h with RSD values below 2%. The extraction recovery rates were assessed by performing triplicate determinations for each sample. The average recovery rates of gallic acid, proanthocyanidin B2, and epicatechin ranged from 93.33% to 101.13%. Thus, this HPLC method demonstrated acceptable specificity, linearity, precision, repeatability, solution stability, and recovery for reliable determination. The validated method was applied to analyze three batches of ethyl acetate extracts for the content of gallic acid, proanthocyanidin B2, and epicatechin, each of which was injected twice. The average contents of the three compounds were measured to be 0.0563%, 0.3707%, and 0.3868% with RSD values of less than 2%.

### 3.3 Composition identification of EAE based on UPLC‐Q‐Exactive Orbitrap‐HRMS

**FIGURE 3:**
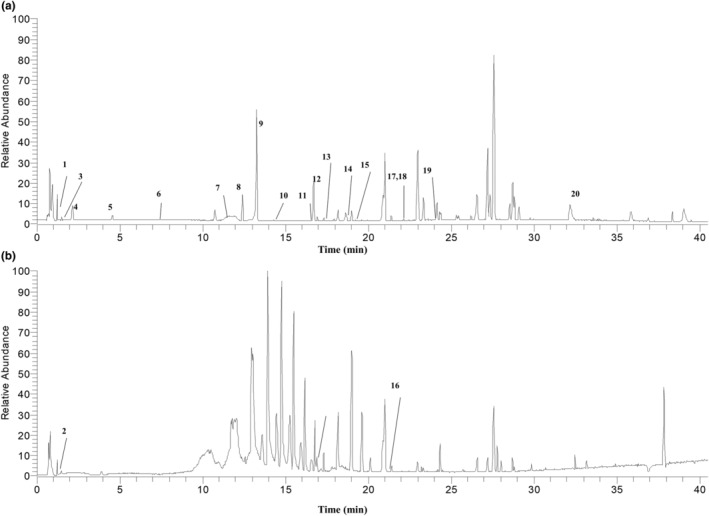
The total ion chromatogram of EAE from *Fagopyrum dibotrys* (D. Don) Hara is at negative mode (a) and positive mode (b). The numbers represent compounds as in Table 1.

From the EAE of golden buckwheat, 20 compounds were confirmed, including nine flavonoids, nine organic acids, one nucleoside, and one alkaloid, of which nine compounds were confirmed with available standards (Figure 3 and Table 1). In this study, a total of 18 compounds were tested in negative mode, while others were captured in positive mode. The analyses conducted in both modes mutually complement each other by facilitating the resolution of chemical structures and enhancing the precision of isomer identification. Further, the fragmentation pathways analysis was carried out.

**TABLE 1:** Identification of chemical constituents of ethyl acetate extract of *Fagopyrum dibotrys* (D. Don) Hara by UPLC‐Q‐Exactive Orbitrap‐HRMS.

No.	*t*_R_ /min	Molecular formula	MS1	[M‐H]^−^	[M + H]^+^	Δ/ppm	Fragment ions	Proposed compound	Reference
1	1.26	C_6_H_8_O_7_	191.0193	191.0192		0.52	173.0084,129.0184,111.0077,102.9478,87.0076	Citric acid	Huang, Liang, Wei, et al. (2021)
2	1.39	C_10_H_13_N_5_O_4_	268.1031		268.1040	−3.36	136.0615,119.0349	Adenosine	Wang et al. (2021)
3	1.49	C_4_H_6_O_4_	117.0183	117.0188		−4.27	99.0076,73.0283,55.0177	Succinic acid	Gao et al. (2021)
4	2.18	C_7_H_6_O_5_	169.0135	169.0137		−1.18	125.0234,107.0129	Gallic acid*	Nie et al. (2015)
5	4.54	C_7_H_6_O_4_	153.0185	153.0188		−1.96	109.0284,91.0177	Protocatechuic acid*	Yu et al. (2016)
6	7.48	C_7_H_6_O_3_	137.0235	137.0239		−2.92	119.0124,108.0206,93.0334	Protocatechualdehyde	Sun et al. (2019)
7	11.44	C_8_H_8_O_4_	167.0343	167.0344		−0.60	152.0107,123.0441,108.0206	Vanillic acid	Ma et al. (2014)
8	12.46	C_30_H_26_O_12_	577.1342	577.1346		−0.69	425.0886,407.0777,289.0722,125.0234	Proanthocyanidin B2*	Zhang, Zheng, et al. (2021)
9	13.23	C_15_H_14_O_6_	289.0722	289.0712		3.46	165.0185,147.0441,139.0392,123.0441	Epicatechin*	Xu et al. (2019)
10	14.47	C_9_H_8_O_3_	163.0393	163.0395		−1.23	119.0492	Hydroxycinnamic acid	Sheng et al. (2018)
11	16.56	C_27_H_30_O_16_	609.1473	609.1456		−2.79	300.0282,271.0252,255.0300,300.0279,271.0252,255.0301,151.0027,107.0126	Rutin*	Nijat et al. (2020)
12	16.73	C_21_H_20_O_12_	463.0858	463.0877		−4.10	300.0310,271.0613,243.0647,227.0333,151.0393	Hyperoside*	Yu et al. (2021)
13	17.36	C_7_H_6_O_3_	137.0235	137.0239		−2.92	93.0334	Salicylic acid	Cui et al. (2020)
14	18.64	C_9_H_16_O_4_	187.0970	187.0970		0	143.1072,125.0962,97.0647,69.0336	Azelaic acid	Huang, Liang, Sun, et al. (2021)
15	19.24	C_15_H_12_O_6_	287.0566	287.0556		3.48	259.0615,243.0664	Eriodictyol	Lin et al. (2021)
16	21.28	C_20_H_17_NO_4_	336.1223		336.1230	−2.08	320.0912,306.0756,292.0962,278.0805	Berberine*	Hao et al. (2020)
17	22.06	C_15_H_10_O_6_	285.0408	285.0399		3.16	175.0396,151.0028,133.0285,107.0129	Luteolin*	Huang, Liang, Wei, et al. (2021)
18	22.11	C_15_H_10_O_7_	301.0359	301.0348		3.65	273.0414,245.0454,178.9980,151.0028	Quercetin*	Yan et al. (2021)
19	23.96	C_15_H_12_O_5_	271.0616	271.0607		3.32	187.0399,177.0186,165.0186,151.0028,119.0492,107.0127	Naringenin	Wang et al. (2021)
20	32.31	C_15_H_10_O_5_	269.0461	269.0450		4.09	241.0508,225.0558,197.0605,181.0652	Genistein	Zhao et al. (2019)

*Note*: * means that the compound has been confirmed by comparison with standard.

#### 3.3.1 Flavonoids

**FIGURE 4:**
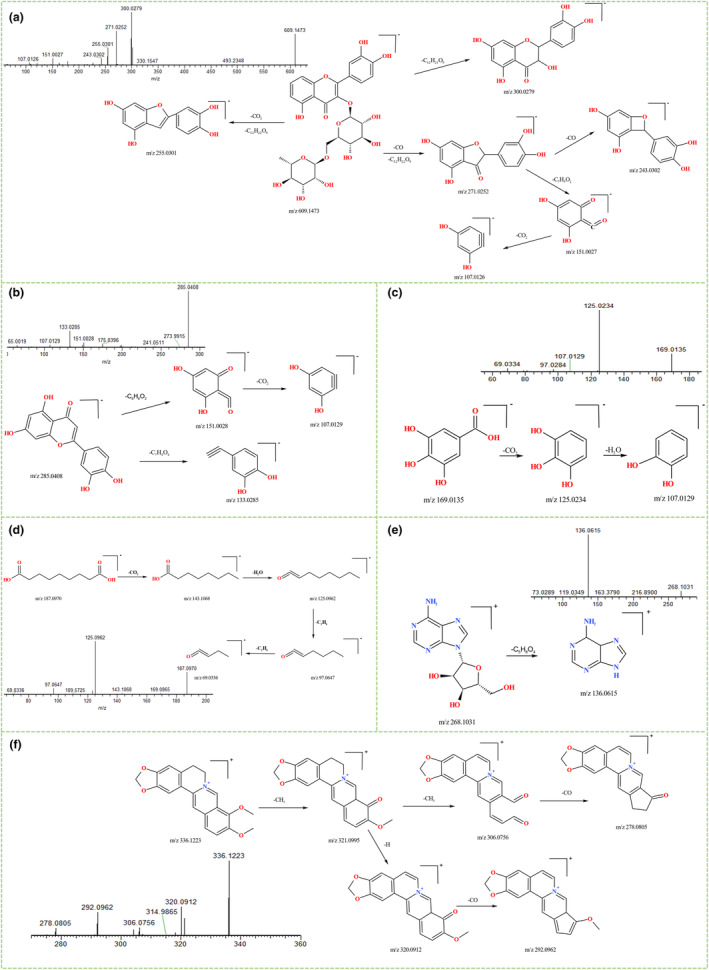
Proposed fragmentation pathways and MS2 of the rutin (a), luteolin (b), gallic acid (c), azelaic acid (d), adenosine (e), and berberine (f).

In this experiment, nine flavonoids were identified from the EAE, of which hyperoside, luteolin, rutin, quercetin, eriodictyol, procyanidin B2, and epicatechin were accurately identified by comparison with standards. These flavonoids are mostly attached to sugars to form glycosides or in the form of carbon glycosyl groups, which are mainly deglycosylated during cleavage, resulting in the release of neutral molecules such as CO, CO_2_, CH_3_, and H_2_O and the formation of a series of characteristic ionic peaks. As an example, the retention time of rutin was 16.39 min and it responded well in the negative mode. After fitting with Xcalibur™ software, the primary MS information revealed a molecular ion peak *m/z* 609.1473, suggesting its molecular formula to be C_27_H_30_O_16_. The secondary mass spectral information can be interpreted as follows: The parent ion *m/z* 609.1473 lost the glycosyl to yield *m/z* 300.0279 (C_15_H_8_O_7_), *m/z* 271.0252 (C_14_H_7_O_6_), and *m/z* 255.0301 (C_14_H_7_O_5_). The ion of *m/z* 271.0252 (C_14_H_7_O_6_) continued cleavage to *m/z* 151.0027 (C_7_H_3_O_4_) or loss of neutral CO to *m/z* 243.0302 (C_13_H_7_O_5_). Finally, *m/z* 151.0027 (C_7_H_3_O_4_) experienced CO_2_ loss, resulting in fragmentation to *m/z* 107.0126 (C_6_H_3_O_2_). The compound was finally confirmed as rutin by comparison with the standard and its possible fragmentation pathway was postulated (Figure 4a) (Nijat et al., 2020).

Luteolin also exhibited positive results in the negative mode of analysis. The primary molecular ion peak of luteolin in MS was observed at *m/z* 285.0408 (C_12_H_10_O_6_) (Huang, Liang, Wei, et al., 2021). The proposed cleavage pathway involved the loss of C_7_H_4_O_4_ and C_8_H_6_O_2_ from the molecular ion to generate fragment ions at *m/z* 133.0285 [M‐H‐C_7_H_4_O_4_]^−^ and *m/z* 151.0028 [M‐H‐ *m/z* 151.0028]^−^, respectively. Subsequently, the fragment ion at *m/z* 151.0028 further underwent cleavage and lost CO_2_, resulting in the formation of a fragment ion at *m/z* 107.0129 [M‐H‐C_8_H_6_O_2_‐CO_2_]^−^ (Figure 4b). It was confirmed as luteolin by comparison with the standard.

#### 3.3.2 Carboxylic acids

Nine carboxylic acids were identified from EAE, among which gallic acid and protocatechuic acid were accurately identified through comparison with control samples. These compounds predominantly underwent losing neutral molecules such as H_2_O, HCOOH, and CO_2_ during the lysis process. Using gallic acid as an example (Nie et al., 2015), the first cleavage of the precursor ion *m/z* 169.0135 (C_7_H_6_O_5_) lost a CO_2_ molecule to become *m/z* 125.0234 [M‐H‐CO_2_]^−^, and then lost the neutral molecule H_2_O to form *m/z* 107.0129 [M‐H‐CO_2_‐H_2_O]^−^ (Figure 4c). Similarly, the precursor *m/z* 187.0970 (azelaic acid, C_9_H_16_O_4_) lost CO_2_, H_2_O, and C_2_H_4_ sequentially through four cleavages to obtain characteristic fragments: *m/z* 143.1068 [M‐H‐CO_2_]^−^, *m/z* 125.0962 [M‐H‐CO_2_‐H_2_O]^−^, *m/z* 97.0647 [M‐H‐CO_2_‐H_2_O‐C_2_H_4_]^−^, and *m/z* 69.0336 [M‐H‐CO_2_‐H_2_O‐C_2_H_4_‐C_2_H_4_]^−^ (Figure 4d) (Huang, Liang, Sun, et al., 2021).

#### 3.3.3 Nucleoside

Here, we successfully identified adenosine, a nucleoside that demonstrated a significant response in positive ion mode (Wang et al., 2021). The primary MS information yielded a molecular ion peak at *m/z* 268.1031, for which the molecular formula was determined as C_10_H_13_N_5_O_4_ through fitting with Xcalibur™ software. It was suggested that the primary ionic structure at *m/z* 268.1031 underwent the loss of a furanose residue, leading to the generation of *m/z* 136.0615 [M + H‐C_5_H_8_O_4_]^+^ (Figure 4e).

#### 3.3.4 Alkaloids

Berberine was identified in the ethyl acetate extract of golden buckwheat. We proposed a cleavage pathway for the parent ion at *m/z* 336.1223 (C_20_H_17_NO_4_), which was subsequently confirmed by comparison with standard samples (Hao et al., 2020). Initially, the primary fragment has removed a CH_3_ group, resulting in the formation of *m/z* 321.0995 [M + H‐CH_3_]^+^. Subsequently, both the discarded CH_3_ and H groups led to the formation of *m/z* 306.0756 [M + H‐CH_3_‐CH_3_]^+^ and *m/z* 320.0912 [M + H‐CH_3_‐H]^+^, respectively. Finally, the fragmentation involved the removal of a CO molecule, giving rise to *m/z* 278.0805 [M + H‐CH_3_‐CH_3_‐CO]^+^ and *m/z* 292.0962 [M + H‐CH_3_‐H‐CO]^+^, separately (Figure 4f).

### 3.4 EAE protected against the histological damage of lung tissues in ALI mice

**FIGURE 5:**
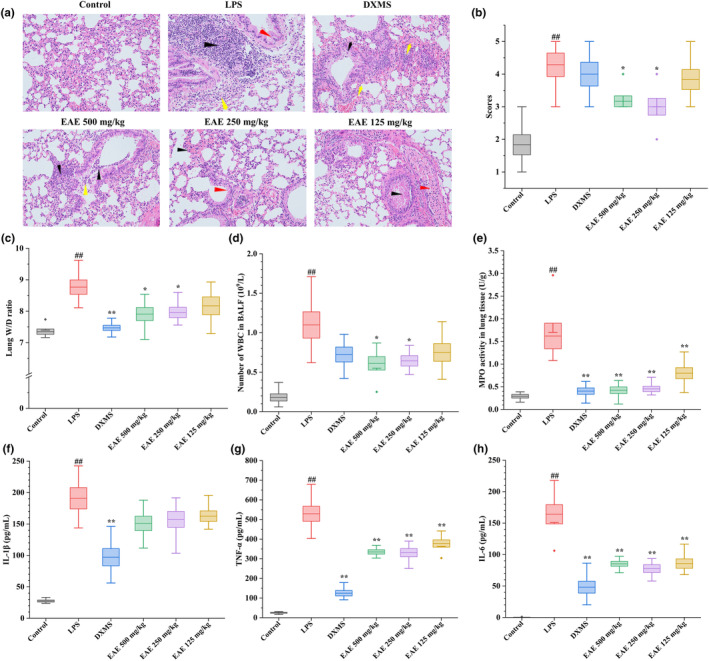
Effects of EAE from *Fagopyrum dibotrys* (D. Don) Hara protect against LPS‐induced ALI in mice. (a) HE pathology testing of lung tissue (200×); (b) Histological inflammation‐associated scores of lung tissue; (c) W/D ratio of lung tissue; (d) production of WBC in BALF; (e) MPO activity in lung tissue; (f) production of IL‐1β in BALF; (g) production of TNF‐α in BALF; (h) production of IL‐6 in BALF. ^#^.01 ≤ *p* < .05 and ^##^ *p* < .01 versus control group; *.01 ≤ *p* < .05 and **p < .01 versus LPS group.

The HE pathology testing was used to evaluate the histological damage of lung tissues. The histological inflammation‐related score of the lung tissue is rated from 0 to 5 on a scale from mild to severe. As shown in Figure 5a,b, the lung of mice in the control group had normal tissue structure, with a non‐thickened alveolar wall, no inflammatory permeability, and no hyperemia. Compared control group, the group exposed to LPS exhibited evident infiltration of lymphocytes and neutrophils, along with the formation of granulation tissue (black arrow). Additionally, there was observed exfoliation of bronchial epithelial cells and necrosis (red arrow), as well as loss of the surrounding alveolar structures (yellow arrow). The histopathological scores of the lung tissues in the LPS group were considerably elevated. However, treatment with dexamethasone and EAE resulted in a reduction of histological inflammation scores in the lungs of the mice with ALI. Notably, the high and medium doses of EAE exhibited a significant decrease in lung histopathology scores compared to the control group. The results suggested that the EAE of golden buckwheat can reduce the degree of pathological damage in the lung tissue of ALI mice.

### 3.5 Effect of EAE on the W/D ratio of lung tissues

The W/D ratio of the lungs was measured to investigate variations in the permeability of pulmonary vasculature to water caused by LPS, and the results are presented in Figure 5c. Apparently, the intervention of LPS caused a remarkable increase in the W/D ratio of the lung tissue (*p* < .01). In comparison to the LPS group, treatment with dexamethasone resulted in a notable reduction of the W/D values in lung tissue. Similarly, the EAE of golden buckwheat demonstrated a dose‐dependent reduction in the W/D values. These findings suggest that the EAE of golden buckwheat could alleviate lung edema in ALI mice, thus mitigating the inflammatory response.

### 3.6 Effects of EAE on the production of WBC in BALF

The number of WBCs in the alveoli serves as a crucial indicator of lung inflammation. The number of WBCs increases when the lungs are exposed to infections, inflammation, or other pathological conditions. Therefore, we conducted tests to measure the number of WBC in the BALF (Figure 5d). The data indicated a significant elevation in WBC count in the BALF of ALI mice. Conversely, the dexamethasone‐treated group demonstrated a potential decrease in WBC count in the BALF, while the reduction in WBC count by EAE was observed to be dose‐dependent.

### 3.7 Effects of EAE on MPO activity in lung tissue

The activity of MPO serves as a biochemical marker for the infiltration of neutrophils and macrophages into the lungs. In Figure 5e, significantly higher MPO activity was observed in the LPS group than control group, indicating increased inflammation (*p* < .01). In contrast, treatment with dexamethasone led to a notable reduction in MPO activity. Furthermore, administration of EAE demonstrated a clear inhibition of MPO activity compared to the LPS group. It suggested that EAE effectively alleviates lung inflammation in ALI mice.

### 3.8 Effect of EAE on the concentrations of IL‐1β, TNF‐α, and IL‐6 in BALF

The levels of IL‐1β, TNF‐α, and IL‐6 in BALF of mice were quantified by Elisa and are reported in Figure 5f–h. The LPS group of mice showed significantly higher expression levels of IL‐1β, TNF‐α, and IL‐6. However, in the DXMS group, the content of IL‐1β, TNF‐α, and IL‐6 in BALF noticeably decreased (*p* < .01) compared to the model group. Moreover, in the three different dose groups of EAE, the concentration of TNF‐α and IL‐6 in BALF of mice in the three different EAE dose groups was significantly lower (*p* < .01) in comparison with the model group. Among them, a dose‐dependent pattern was observed in the 125 and 250 mg/kg dose groups. Although the concentration of IL‐1β showed a tendency to decrease, the difference was not significant in terms of statistical significance. The data indicated that the EAE component derived from golden buckwheat possessed the ability to decrease the production of IL‐1β, TNF‐α, and IL‐6 in the BALF of ALI mice. This subsequently led to the amelioration of lung inflammation and attenuation of the inflammatory response.

### 3.9 Effect of EAE on LPS‐induced TLR4/NLRP3 signaling pathway

**FIGURE 6:**
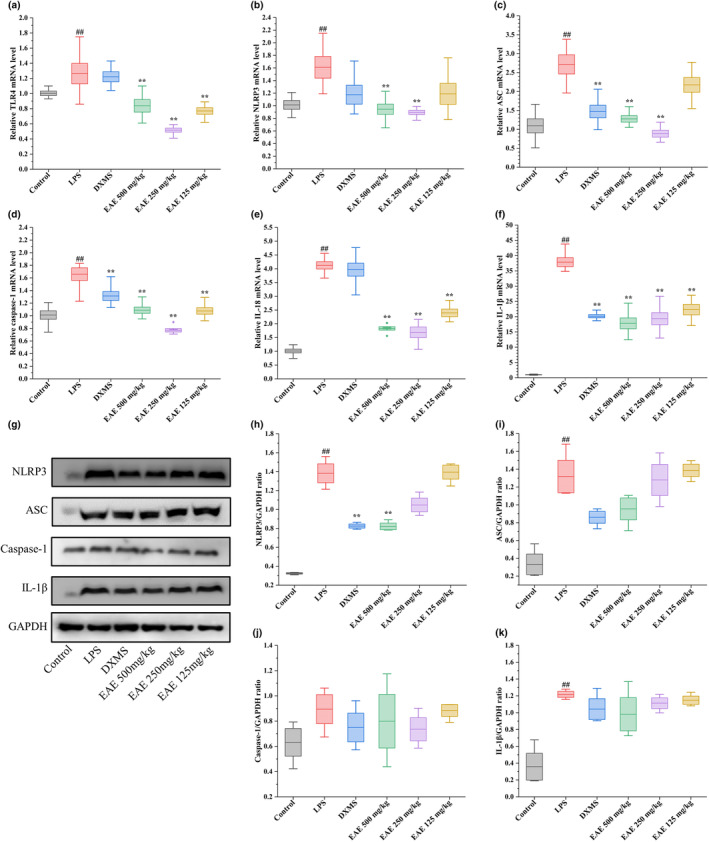
Effects of EAE from *Fagopyrum dibotrys* (D. Don) Hara on TLR4/NLRP3 pathway‐related proteins and mRNAs in LPS‐induced ALI mice. (a–f) represent the effects of EAE on TLR4 (a), NLRP3 (b), ASC (c), caspase‐1 (d), IL‐18 (e), and IL‐β (f) mRNA levels in mouse lung tissue, respectively. (g) and (h) represent the effects of EAE on NLRP3 (h), ASC (i), caspase‐1 (j), and IL‐1β (k) protein expression in mouse lung tissues, respectively. ^#^.01 ≤ *p* < .05, ^##^ *p* < .01 versus control group; *.01 ≤ *p* < .05, **p < .01 versus LPS group.

In this part, we aimed to investigate the impact of EAE on the TLR4/NLRP3 pathway. The gene levels of TLR4, NLRP3, ASC, caspase‐1, IL‐18, and IL‐1β were assessed using real‐time qPCR. As illustrated in Figure 6a–f, the expression of these mRNAs was elevated due to LPS interference, indicating the upregulation of the TLR4/NLRP3 signaling pathway by LPS. Remarkably, treatment with dexamethasone led to a significant reduction in ASC, caspase‐1, and IL‐1β mRNA levels in lung tissues (*p* < .01). Moreover, the administration of EAE derived from golden buckwheat effectively suppressed the mRNA levels of TLR4, NLRP3, ASC, caspase‐1, IL‐18, and IL‐1β in the lung tissues of ALI mice induced by LPS in a partially dose‐dependent manner. Furthermore, we conducted western blot analysis to check the relative expression quantities of the proteins (Figure 6g–k). The administration of LPS resulted in a notable increase in the expression of NLRP3, ASC, caspase‐1, and IL‐1β proteins. However, this upregulation was effectively inhibited by treatment with EAE, specifically in the 500 and 250 mg/kg dose groups, as well as by dexamethasone treatment. Notably, the inhibition of NLRP3 by both EAE and dexamethasone was found to be evident statistically (*p* < .01). These findings collectively suggest that EAE exhibits a preventive effect against ALI induced by LPS, partially mediated by the downregulation of the NLRP3 pathway.

## 4 DISCUSSION

ALI is a disease characterized by an exaggerated inflammatory response in the lungs leading to impaired gas exchange and respiratory distress, with high morbidity and mortality (Long et al., 2022; Sun & Li, 2022). The TLR4/NLRP3 pathway is critical in the pathogenesis of ALI, causing sustained production of cellular factors such as IL‐1β and IL‐18, which ultimately leads to a massive outbreak of lung inflammation, resulting in tissue injury and even ARDS (Meyer et al., 2021; Wang et al., 2020). Therefore, aiming at the TLR4/NLRP3 pathway holds promise as a therapeutic strategy for managing ALI by attenuating the exaggerated inflammatory response and reducing lung injury. Golden buckwheat is an important plant with a dual role as a highly nutritious food source and as a remedy for respiratory diseases. Our comprehensive review highlighted the consistent body of pharmacological evidence supporting the remarkable therapeutic properties of golden buckwheat, including its anti‐inflammatory, anticancer, antioxidant, antibacterial, and antidiabetic effects (Zhang, He, et al., 2021). These noteworthy findings collectively indicate the utility of golden buckwheat as a promising beneficial source for alleviating ALI.

Stimulation of macrophages with LPS results in the release of several important molecules, including NO, IL‐1β, and IL‐6. As a potent immune mediator, NO had a critical role in helping to regulate immune response, including activation and recruitment of immune cells. In addition, IL‐1β and IL‐6 are inflammatory factors that are released in response to LPS stimulation. These molecules are key players in mediating the inflammatory cascade, promoting immune cell activation, and initiating defense mechanisms against pathogens. Research has shown that golden buckwheat extract could delay the development of inflammation in rats with osteoarthritis of the knee by inhibiting the production of IL‐1β, TNFα, and IL‐6 (Pan & Ji, 2021). Chen et al. (2022) found that golden buckwheat extract can alleviate oxidative stress and inflammatory response, thereby improving ALI in paraquat‐poisoned rats. Another study reported that gold buckwheat aqueous can treat ARDS rats (Wang et al., 2023). However, comprehensive studies on various extracts of golden buckwheat regarding their anti‐inflammatory activity and the identification of superior anti‐inflammatory components are insufficient. With the intention of systematically explore the pharmacodynamic material basis of the anti‐inflammatory activity of golden buckwheat, the secretion of NO, IL‐1β, and IL‐6 in LPS‐stimulated RAW 264.7 were analyzed by six extracts of golden buckwheat in this study. Our results showed that except for AE, the other five extracts of golden buckwheat could significantly inhibit the production of these inflammatory factors at a working concentration of 40 μg/mL, and EAE exhibited the strongest anti‐inflammatory activity among them. Therefore, EAE was confirmed for subsequent chemical composition analyses and in vivo studies of anti‐inflammatory mechanisms.

UPLC‐Q‐Exactive Orbitrap‐HRMS is an advanced analytical instrument renowned for its exceptional chromatographic resolution and separation efficiency (Dong et al., 2021). These features make it well‐suited for the precise and targeted analysis of specific analytes with low limits of detection, and the results obtained will assist in a deeper comprehension of the chemical components of plant‐derived medicines and their potential therapeutic properties. Based on the accuracy and sensitivity of the UPLC‐Q‐Exactive Orbitrap‐HRMS method, as well as the high‐resolution MS data provided, our study accurately identified and characterized a total of 20 chemical components in EAE, including nine flavonoids, nine organic acids, one nucleoside, and one alkaloid, which were crucial for further research on the therapeutic potential and pharmacological activities of EAE. Beyond the specific analysis of EAE, an additional valuable outcome of our research is the insight obtained regarding the fragmentation modes of the identified compounds. By examining the MS analysis of the fragmentation structures, we can provide valuable materials for the structural characterization of similar substances in other kinds of plants. In addition, we have developed a robust assay method to precisely determine the quantity of gallic acid, proanthocyanidin B2, and epicatechin in EAE. By accomplishing the quantification of these key active components, we were able to effectively evaluate the stability of the extract and ensure quality control and monitoring. It not only contributed to the reliable assessment of golden buckwheat EAE's chemical composition but also held significance for its future research, development, and application in various fields, including functional food development, nutraceuticals, and herbal medicine formulations.

In this study, we found that EAE could downregulate the inflammatory response, attenuate pathological damage and the tissue edema in lung tissue in LPS‐induced ALI mice. We also observed that EAE was effective in suppressing the number of WBCs, attenuating MPO activity, and suppressing the cytokines generation (IL‐1β, TNFα, and IL‐6), especially at doses of 500 and 250 mg/kg. Furthermore, we investigated the mechanisms by which EAE controls inflammation at the gene and protein perspective. Our research found that EAE downregulated TLR4, NLRP3, ASC, caspase‐1, IL‐18, and IL‐1β mRNA and protein expression. This means that EAE's inhibition of the ALI lung inflammatory response may be a consequence of the regulation of the TLR4/NLRP3 signaling pathway. The flavonoids in golden buckwheat were the main contributors to the anti‐inflammatory effects, according to our previous report. As a type of flavonoid, proanthocyanidin B2, present at 0.3707% in EAE, has been shown to alleviate paraquat‐induced ALI rat by preventing activation of NLRP3 (Jiang et al., 2018). Additionally, it has been found to suppress LPS‐induced inflammation and apoptosis in human alveolar epithelial cells (AECs) and lung fibroblasts (LFs) by inhibiting NF‐κB and NLRP3 inflammasome in vitro (Jiang et al., 2020). Epicatechin has been observed to down‐regulate the MAPK/NF‐κB pathway, thereby protecting LPS‐induced ALI in mice (Li et al., 2022; Xing et al., 2019). Luteolin exhibits various mechanisms to alleviate ALI, including the activation of Treg/IL‐10 to alleviate caspase‐11‐dependent pyroptosis (Zhang, Zhang, et al., 2021), activation of epithelial sodium channels via the cGMP/PI3K pathway (Hou et al., 2022), activation of ERK1/2‐ and Ca^2+^‐dependent HO‐1 induction (Park et al., 2018), and inhibition of Akt/NFκB, MAPK/NFκB, MEK/ERK, and PI3K/Akt pathways (Kuo et al., 2011; Lee et al., 2010; Li et al., 2012). Furthermore, organic acid might also contribute to anti‐inflammatory benefits. Gallic acid has been reported to be able to improve ulcerative colitis in mice by suppressing NLRP3 inflammasome (Yu et al., 2023) and alleviate gouty arthritis by enhancing Nrf2 signaling and repressing NLRP3 inflammasome activation and cleavage (Lin et al., 2020). Although direct evidence of gallic acid's therapeutic effect on ALI is lacking, these studies emphasize its potential in suppressing NLRP3 inflammasome which provides basses for its application in ALI treatment. Apparently, multiple compounds present in EAE implicated in the inhibition of lung inflammatory response in ALI models, but their potential synergistic effects between these components still need further exploration.

## 5 CONCLUSION

Golden buckwheat is officially recognized as a protective food in China. As well as being consumed as a healthcare food, it can also be used in the livestock industry. The present research demonstrated that the EAE extraction from golden buckwheat exhibited the most potent in vitro anti‐inflammatory effect. EAE contains 20 compounds, primarily consisting of flavonoids and organic acids. Through inhibition of the inflammatory response in the ALI mice model induced by LPS, EAE demonstrated a protective effect against ALI, possibly via the down‐regulation of the TLR4/NLRP3 signaling pathway by the various compounds present in EAE. Our findings provide compelling evidence supporting the utilization of golden buckwheat as a source of functional food for the prevention or treatment of ALI as a promising approach.

## AUTHOR CONTRIBUTIONS

**Yingfan Hu:** Conceptualization (lead); data curation (equal); investigation (equal); writing – original draft (lead); writing – review and editing (lead). **Xiaomin Liu:** Data curation (lead); investigation (lead); methodology (lead); writing – original draft (equal). **Yu Song:** Conceptualization (equal); data curation (supporting); investigation (supporting); methodology (equal). **Yan Zhang:** Investigation (equal); methodology (supporting); writing – review and editing (lead). **Wei Li:** Investigation (supporting); methodology (equal). **Lele Zhang:** Investigation (supporting); methodology (supporting). **Anqi Wang:** Investigation (supporting); methodology (supporting). **Qian Su:** Investigation (supporting); methodology (supporting). **Zhiyong Yang:** Investigation (supporting); methodology (supporting). **Liang Zou:** Funding acquisition (lead); resources (lead); supervision (lead); writing – review and editing (equal).

## FUNDING INFORMATION

The research outputs of this Open Research Project Program are funded by the Science and Technology Development Fund (SKL‐QRCM (UM)‐2023‐2025) and the State Key Laboratory of Quality Research in Chinese Medicine, University of Macau (No. SKL‐QRCM‐OP23019). This work was also supported by the Sichuan Science and Technology Program (2021YFH0169), the Key Discipline Program of Chengdu Municipal Health Commission‐Clinical Pharmacy (ZD‐01‐06), and the Chengdu Medical Research Project (2021052, 2021248).

## CONFLICT OF INTEREST STATEMENT

None.

## ETHICAL APPROVAL

Animal experiments in the study were conducted in accordance with the Guidelines for Care and Use of Laboratory Animals of Chengdu University and approved by the Animal Ethics Committee of Chengdu University.

## Data Availability

The data that support the findings of this study are available on request from the corresponding author. The data are not publicly available due to privacy or ethical restrictions.
